# Sickle cell disease and COVID‐19: Atypical presentations and favorable outcomes

**DOI:** 10.1002/jha2.74

**Published:** 2020-08-04

**Authors:** Marie‐Agnès Azerad, Firas Bayoudh, Thierry Weber, Jean‐Marc Minon, Olivier Ketelslegers, Marie Hoyoux, Xueying Ren, Olivier Kaye, Nils De Marneffe, Vincent Fraipont, Catherine Masset, Anne Sophie Bouillon, Aurélie Jaspers, Malek Tebache, Guillaume D'Hoen, Anousha Habibi, André Efira, Jecko Thachil, Hans Deckmyn, Yves Beguin

**Affiliations:** ^1^ Department of Haematology CHU of Liège at site CHR Citadelle Liège Belgium; ^2^ Thierry Weber Head of pneumology department CHR Citadelle Liège Belgium; ^3^ Head of Department of Laboratory Medicine Thrombosis‐haemostasis and Transfusion unit CHR Citadelle Liège Belgium; ^4^ Department of Laboratory Medicine, Hemopathology Unit CHR Citadelle Liège Belgium; ^5^ Department of Paediatrics CHU of Liège at site CHR Citadelle Liège Belgium; ^6^ Department of Internal Medicine CHR Citadelle Liège Belgium; ^7^ Department of Rheumatology CHR Citadelle Liège Belgium; ^8^ Department of Cardiology CHR Citadelle Liège Belgium; ^9^ Head of Intensive Care Unit CHR Citadelle Liège Belgium; ^10^ Department of Nephrology and dialysis CHR Citadelle Liège Belgium; ^11^ Department of Radiology CHR Citadelle Liège Belgium; ^12^ Medical Direction Scientific Committee for COVID ‐19 at CHR Citadelle Liège Belgium; ^13^ Unité des Maladies Génétiques du Globule Rouge, APHP, Hôpitaux Universitaires Henri‐Mondor, UPEC, Institut Mondor de recherche biomedicale (IMRB) Institut National de la Santé et de la Recherche Medicale (INSERM) U955 DHU A‐TVB Créteil France; ^14^ Department of Haematology CHU Brugmann Bruxelles Belgium; ^15^ Department of Haematology Manchester Royal Infirmary Manchester UK; ^16^ Laboratory for Thrombosis Research KU Leuven Campus Kulak Kortrijk Kortrijk Belgium; ^17^ Department of Haematology, CHU of Liège and GIGA I3 University of Liège Liège Belgium

COVID‐19 pandemic has already claimed several lives. Individuals with underlying health conditions including sickle cell disease (SCD) are considered to be at higher risk of complications from the severe acute respiratory syndrome coronavirus 2 (SARS‐CoV‐2). Here, we report our experience with three patients with severe SS SCD, all originating from Congo that contracted COVID‐19 infection (Table 1). All three developed only mild symptoms with few respiratory symptoms at presentation that did not require intensive care unit admission. All three of them initially received automated red cell exchange transfusion (ET) according to the current recommendations [1].

**TABLE 1:** Characteristics of the patients

Patient	1	2	3
Age	23	44	23
Sex	Female	Male	Female
Blood group	A Positif ccee Kell négatif	AB Positif ccee Kell négatif	A Négatif ccee Kell négatif
Hemoglobinopathy	SS (Bantu phenotype)	SS (Bantu phenotype) and Alpha Thalassemia trait	SS (Bantu phenotype) and G6PD deficiency
Origin	Congo	Congo	Congo
HTA	No	Yes	No
Obesity	No	No	No
Hydroxyurea	Yes low Compliance	Yes	Yes
Aspirin	Yes	No	No
Regular exchange therapy	Yes	No	No
Deferasirox	No	No	Yes
COVID ‐19 diagnosis	PCR	PCR	PCR
Lung lesion at entry	Unilateral ground glass	Mild typical COVID lesion	Pneumomediastin ; no lung lesion
Length of PCR positivity			> 30 days
Respiratory symptoms	Cough	Cough	None
Others symptoms	Abdominal pain, weight loss	Leg ulcer	Dehydration after vomiting
Deep venous thrombosis	No	No	localized DVT catheter site 11 days after ET
Renal insufficiency	No	Yes but already basal	Acute and reversible
Intensive care	No	No	No
Exchange therapy	Regular, one during COVID	2 during COVID	1 ( suspected DHTR)
Hydroxychloroquin	No	400 mg x 2/d then 200 mg x2/d 5 d	No
Anticoagulants	No	No (renal insufficiency)	enoxaparin 40 mg/d prophylactic, then after DVT tinzaparin 175 U/kg

Our first patient is a young woman with SS Bantu phenotype, aged 23, who came for a regular visit for planning her next ET. Her usual treatment regimen was ET using six red blood cell units (RBCU) every 5 weeks with a target HbS fraction of 50% for previous recurrent painful vaso‐occlusive crises (VOC). She was also receiving treatment with hydroxycarbamide but with poor compliance, folic acid, and aspirin to maintain her arteriovenous fistula. She mentioned having had cough 2 weeks prior to the current visit and a fever of 38°C treated by her general practitioner with amoxicillin for 7 days with good response except for some mild VOC symptoms. She was screened positive for COVID‐19 by PCR nasopharyngeal swab. Computed tomography (CT) of her thorax showed unilateral ground glass features suggestive of COVID‐19. She was hospitalized for 48 h. ET was performed the following day and also treated with clarithromycin for 7 days with very good outcome and no need for re‐hospitalization.

The second patient is a 44 year‐old man with SS SCD with very few past episodes of VOC but with history of hypertension and severe renal insufficiency with significant proteinuria. He had basal hemoglobin (Hb) around 5.4 g/dL despite weekly erythropoietin (EPO) administration and poor compliance with low‐dose hydroxyurea and ACE inhibitors. He came for a routine visit with a symptomatic leg ulcer for 2 weeks, which he linked to hydroxyurea that he had been more compliant with at a low dose of 500 mg daily. He also mentioned coughing and slight dyspnea for about a week. He was tested SARS‐CoV‐2 positive by PCR on the nasopharyngeal swab. His chest CT scan showed typical features of COVID infection. After a transfusion of two RBCUs, an isovolumic ET with an HbS target fraction of 20%, and a target Hb of 7 g/dL was also performed. He also received hydroxychloroquine while an in‐patient according to our local practice. About 10 days after admission, he developed abdominal pain with ascites and hepatic enlargement. At this time, quite surprisingly his Hb level rose up to 10 g/dL, with extreme reticulocytosis. A second ET was performed and the weekly EPO injection was with‐held. He improved well with healing of his leg ulcer and rapid clearance of his lung lesion but with no impact on his renal function (creatinine clearance under 20 mL/min/1.73²). At day 30, he had normal vital signs; the PCR on the swab was negative, COVID serology still positive. CRP returned to normal, and a repeat CT thorax showed early changes of fibrosis.

**FIGURE 1:**
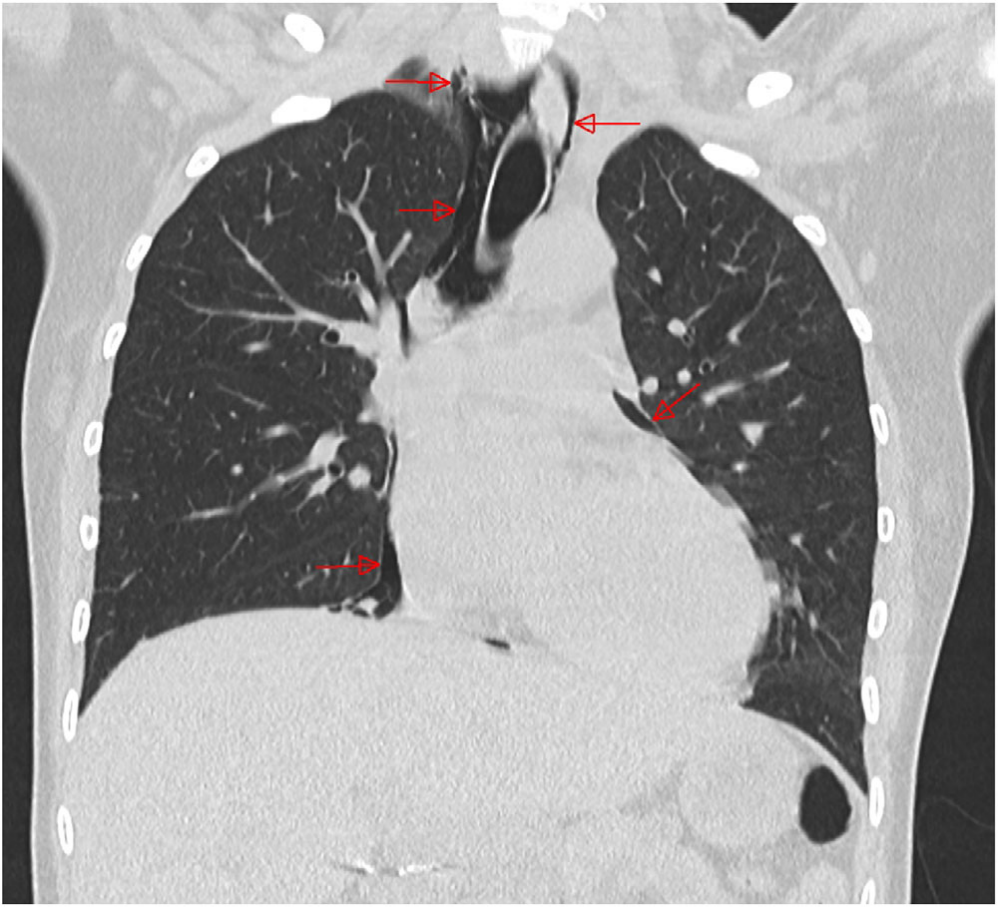
CT scanner showing Spontaneous pneumomediastin with no underlying lung lesion in patient 3 with severe SS sickle cell disease at presentation

The third patient is a 23‐year‐old lady with SS SCD admitted for VOC that developed after repeated bouts of vomiting and developed acute renal insufficiency. Her chest CT revealed a pneumomediastinum with no underlying lung lesions(Figure 1). Following the description of a similar case in the literature [2], she was screened for COVID‐19 by nasal swab and was found to be positive. There were no abnormalities of the gastrointestinal tract on endoscopy. She was receiving treatment with hydroxycarbamide, folic acid, vitamin D, and deferasirox. She received ET and was also treated with ceftriaxone and metronidazole to prevent mediastinitis. While the control chest CT scanner on day 9 showed no more pneumomediastinum and no lung embolism or infiltrate, she developed a focal non occlusive deep venous thrombosis (DVT) on femoralis comunis vena at the site of catheter insertion at day 11 (despite thrombo‐prophylaxis with low dose enoxaparin). A cardiac echography was performed the same day and did not show any indirect sign of lung embolism, an angioscanner performed a few days later did not show any pulmonale embolism. She was anticoagulated with therapeutic low molecular weight heparin and sent home on day 16. She came back 1 week later for severe VOC symptoms. Her chest scan then showed bi‐basal lung lesions atypical for COVID, which could also be interpreted as a delayed hemolytic transfusion reaction (DHTR). SARS‐CoV‐2 test was still positive on nasal swab. Coombs test was negative but since she had been recently transfused, evaluation for the risk of DHTR was made according to the proposed model by the team of Habibi et al [3] and considered intermediate. We decided not to transfuse nor perform a second ET and rather observed her carefully. She had a favorable clinical course, although her Hb level dropped significantly to 5.6 g/dL. Her Hemoglobin spontaneously slowly recovered without any erythropoietin or rituximab adjunction. She received no specific COVID‐19 treatment except oxygen support and antibiotherapy with ceftriaxone with oxygen dependency initially but improved well and was discharged at day 51.

There are now more data in the literature of SCD patients encountering COVID‐19 infections [4, 5, 6]. In the English case reports as in the largest registry to date, the French registry published recently, the authors underline that SARS‐COV2 does not seem to carry an increased risk of mortality or morbidity in patients with SCD [4, 6]. Age was retained as the main risk factor, and patients with the most severe outcome were surprisingly observed in patients with SC phenotype. In our patients, homozygous SS of the most severe Bantu phenotype, there were no features of acute chest syndrome despite COVID‐19 infection and even a pneumomediastinum in the third case.

Based on our short case series, we hypothesized the milder clinical presentation of COVID‐19 may be linked to the sickled red cell. A recent report of the WHO underlines that globally Africa is the least affected with 1.5% of the world's reported cases of COVID‐19 and 0.1% of the world's death [7]. On the contrary, world map repartition of hemoglobinopathies and malaria coincide strongly and WHO estimates that SCD accounts for up to 15% of mortality in Africa, where most children do not live over the age of 5 years despite exciting new therapeutic possibilities, however not reaching low income countries [8]. SCD is due to the modification of a single gene on chromosome 11, which will lead to a loss of membrane deformability of the red blood cell, increased RBC‐endothelial cell adhesion, and development of severe systemic vasculopathy. Endothelial activation plays a central role in the pathophysiology of vaso‐occlusion in SCD along with a now well acknowledged discharge of microparticles and release of free hemoglobin [9]. Resistance to malaria of patients carriers of hemoglobinopathies is a complex phenomenon that has been linked to adaptation of the host response with parasite sequestration and cytoadherence to endothelium [10]. The Koehl group has recently demonstrated by proteomic analysis that neutrophils in SCD present an unexpected activation of the interferon‐α signaling pathway [11]. Gaertner and Massberg, in their fascinating review on immunothrombosis, have also advocated that fibrin forms a trap for invading pathogens and provides the recruitment of immune effector cells, a now recognized important defence mechanism in innate immunity while uncontrolled immunothrombosis can lead to disseminated intravascular coagulation [12]. Morera et al have also shown that erythrocytes may have a direct role in the immune response [13] while Dragovich et al recently demonstrated that cell membrane tension and stiffness might be the dominant defense against Ebola attachment to host cell in an atomic force model [14]. Could this play a role here in the adaptation of the immune response of SCD patients to COVID‐19? This still remains to be further evaluated.

Our patients were treated for their disease with various chronic medications with a relative compliance: all three received hydroxyurea, folic acid, and vitamin D. Patient 1 was treated by chronic ET, whereas patient 3 also received deferasirox until admission. Hydroxyurea has been used for more than 10 years in Europe and has changed the prognostic of sickle cell patients [15]. Hydroxyurea not only augments both HbF level and red blood cell deformability but also maintains the endothelium and Neutrophils Extracellular Traps (NET) equilibrium, and impacts the release of microparticles [16]. In the French registry, in patients with the worst outcome, age was mentioned as the most relevant risk factor but the mean dosage of Hydroxurea in those patients was also low and could have been a confounding factor [6]. Vitamin D deficiency may be correlated with severity of SARS‐Cov 2 infections especially in ethnic minorities [17], however the vitamin D levels of our patients were, despite supplementation, still remarkably low, and thus likely have not played any protective role here.

This short case series shows that the evolution of sickle cell patients with COVID‐19 can be misleading. Our third patient presented initially no lung lesions despite a severe pneumomediastin but after het exchange transfusion developed abnormality on CT scanner compatible with COVID‐19, in fact related to delayed hemolytic transfusion reaction. We encourage clinicians to treat SCD patients with COVID‐19 infection on a case by case basis and not consider them for transfusion invariably but if transfused, to always consider and score for the risk of DHTR [3]. We are hopeful that a careful analysis of the large registries of COVID‐19 among patients with hemoglobinopathies will allow us to better understand this intriguing relationship between COVID‐19 and SCD.
